# Quadratus Lumborum Block versus Fascia Iliaca Compartment Block for Acetabular Fracture Surgery by Stoppa Method: A Double-Blind, Randomized, Noninferiority Trial

**DOI:** 10.1155/2024/3720344

**Published:** 2024-01-05

**Authors:** Alireza Mirkheshti, Morteza Hashemian, Dariush Abtahi, Sara Shayegh, Alireza Manafi-Rasi, Shahram Sayadi, Elham Memary, Nazli Karami, Baharak Rostamian, Alireza Shakeri

**Affiliations:** ^1^Anesthesiology Research Center, Shahid Beheshti University of Medical Sciences, Tehran, Iran; ^2^Department of Anesthesiology and Pain Medicine, Kerman University of Medical Sciences, Kerman, Iran; ^3^Department of Orthopedic Surgery, Shahid Beheshti University of Medical Sciences, Tehran, Iran

## Abstract

**Background:**

Acetabular fracture surgeries are frequently accompanied by protracted and severe perioperative pain, and there is no consensus on optimal pain relief management.

**Aim:**

This study aimed at comparing the analgesic efficacy of fascia iliaca compartment block (FICB) and quadratus lumborum block (QLB) in patients with acetabular fractures undergoing surgery using the Stoppa method.

**Methods:**

In this double-blind, randomized, noninferiority clinical trial, adult patients undergoing spinal anesthesia for acetabular fracture surgery, in Imam Hossein Hospital, Tehran, Iran (IRCT20191114045435N1), were randomly divided into two groups: FICB (*n* = 22) and QLB (*n* = 24). The visual analog scale (VAS) was used to assess the pain intensity at different times for all participants. In addition, the dose of fentanyl required to induce the patient to sit for spinal anesthesia and the pain intensity were evaluated. Moreover, the duration of analgesia and the total amount of morphine consumed in the first 24 h following surgery were evaluated, analyzed, and compared between the two study groups.

**Results:**

FICB and QLB demonstrated effective comparative postoperative analgesic profiles following acetabular fracture surgery; however, no significant differences in VAS values were observed between the two groups during the study. FICB experienced reduced cumulative fentanyl consumption during spinal anesthetic placement, whereas QLB had a significantly lower total morphine demand in the initial postoperative 24 h period.

**Conclusion:**

The lateral QLB and FICB can be introduced as effective routes for analgesia in acetabular fracture surgery using the Stoppa method. *Clinical Trial Registration*. The study was prospectively registered in the clinical trials registry system, on 2021-02-17, with registration number: IRCT20191114045435N1.

## 1. Background

Hip fractures are painful orthopedic emergencies [1]. A relatively uncommon type of hip fracture, acetabular fracture (AF), affects approximately three per 100,000 patients annually [2]. This fracture frequently results from high-energy injuries, including falls from height or a road traffic collision, and requires surgery to stabilize the hip joint and restore hip anatomy [3]. AF is commonly associated with protracted and severe postoperative pain; however, no consensus exists on pain management. On the other hand, uncontrolled pain can raise the risk of delirium, anxiety, and fear; thus, pain management is essential for optimal care in these patients [4].

Pain control in these patients is traditionally based on systemic opioids [4, 5]. Although the use of opioids has been a significant revolution in anesthesia and postoperative pain management, evidence suggests that they can not only produce a variety of adverse effects during the perioperative period but also alter long-term outcomes and have a significant impact on patient's lives, such as the development of opioid dependence or opioid-induced hyperalgesia [6]. Consequently, it is necessary to limit the use of opioids and substitute them with safer and more effective alternatives, such as peripheral nerve block [7].

The pain associated with acetabular fracture surgeries can be managed with regional anesthesia methods such as fascia iliaca compartment block (FICB) or the pericapsular nerve group (PENG) block. These blocks have been noted for their low risk and moderate analgesic efficacy [8, 9]. Furthermore, the quadratus lumborum block (QLB) is a novel plane block that provides satisfactory analgesia after abdominal surgeries such as inguinal hernia repair, laparotomy, and cesarean section [7].

The QLB, initially introduced by Blanco in 2007, is an interfacial plane block situated in the posterior abdominal wall [10]. The pivotal anatomical structures associated with this block are the quadratus lumborum muscle and the thoracolumbar fascia (TLF) [11]. QLB, as a novel truncal regional block technique, shows promise in alleviating both somatic and visceral pain following abdominal surgery [12]. This fascial plane block targets the thoracolumbar nerves by administering local anesthetics around the quadratus lumborum muscle [7]. Various approaches exist for the QL block, including lateral, posterior, and anterior QLB, each applied based on the injection site and with distinct mechanisms tailored to specific operations. Recent case studies have highlighted QLB's analgesic impact on the hip joint [13], confirming its efficacy [14]. The injectate pathway of anterior (or transmuscular) QLB may extend to the paravertebral (PVB) space, providing sensory innervation coverage to the hip nerves [7]. In addition, this block offers the advantage of minimizing quadriceps weakness [15].

Another case study has recently shown that QLB can provide effective analgesia following total hip arthroplasty [13]. To our knowledge, however, no study has investigated the possible analgesic effects of QLB block in acetabular fracture surgery using the Stoppa method.

To this end, in this study, the effects of QLB and FICB on the amount of fentanyl consumed for painless positioning to perform spinal anesthesia in a seated position, the total amount of morphine supplied in 24 h, and the pain VAS score in patients after acetabular fracture surgery utilizing the Stoppa method were evaluated. The null hypothesis was that there were no differences in analgesic efficacy between fascia iliaca compartment block (FICB) and quadratus lumborum block (QLB) in patients with acetabular fractures undergoing surgery.

## 2. Methods

### 2.1. Patients and Study Design

This double-blind, randomized, noninferiority trial was registered with registration number IRCT20191114045435N1 on the clinical trials registry system on February 17, 2021. This research was conducted between August 2020 and March 2021 at Imam Hossein Hospital, Tehran, Iran. Patients eligible for acetabular fracture surgery between the ages of 20 and 70 with ASA classes I and II met the inclusion criteria. This study excluded patients with a history of psychiatric illness, drug addiction, or a body mass index (BMI) of greater than 30 kg/m^2^. In addition, patients were excluded from the study if the plan for spinal anesthesia was changed to general anesthesia during surgery, if they bled more than 1 liter, if the surgery lasted more than 3 hours, if they experienced orthopedic complications during surgery, or if the surgical plan changed. Before the commencement of the study, all patients provided written consent to participate in the survey and to have the results made public. This research was approved by the Shahid Beheshti University of Medical Sciences Ethics Committee and adhered to the ethical principles of the Declaration of Helsinki [16].

Forty-six patients with acetabular fractures were randomly divided into groups A (patients who received FICB) and B (patients who received QLB). A blind anesthesia assistant utilized a computerized random number generator to conduct randomization. Randomization sequences were delivered to the anesthesiologist, who performed the blocks in opaque, sealed envelopes. Based on our previous research, the minimum sample size for each group was 18, with a confidence level of 0.05, a standard deviation (SD) of 55, and a statistical power of 90%. A 30% difference was assumed in average analgesia duration between the two groups [17].

### 2.2. Preparing the Patient before Performing the Block

Before blocking, patients were moved to the block room. After administering 5 ml/kg of intravenous crystalloid liquid, 1 *μ*g/kg of fentanyl, 0.02 mg/kg of midazolam, and 7 L/min of oxygen through a face mask, they were ready to perform the block under standard monitoring.

### 2.3. Fascia Iliaca Compartment Block Procedure

**Figure 1:**
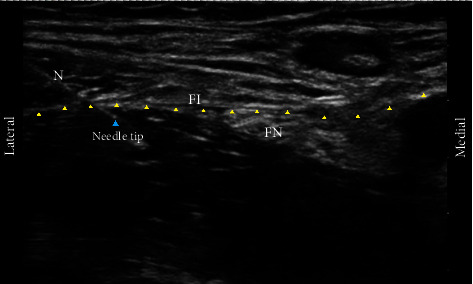
Fascia iliaca compartment block anatomy. FA: femoral artery; FN: femoral nerve; FI: fascia iliaca; N: needle.

FICB was performed after topical anesthesia with 2 mL of 1% lidocaine infiltration when the skin was sterilized with chlorhexidine. Under the direction of a high-frequency linear probe (6–15 MHz/linear array/6 cm scan dept FUJIFILM SonoSite Inc., Tokyo, Japan) ultrasound device (S-nerve; FUJIFILM SonoSite Inc., Tokyo, Japan) that was horizontally aligned in the inguinal region, 0.3 mL/kg of 0.5% ropivacaine was injected by in-plane technique between the iliopsoas muscle and iliac fascia using a needle (B. Braun needle, 22 G, 80 mm, Stimuplex Ultra 360) (Figure 1).

### 2.4. Quadratus Lumborum Block Procedure

**Figure 2:**
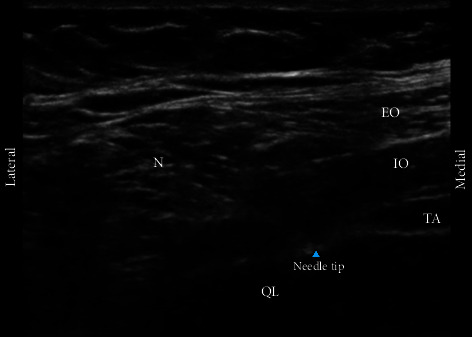
Lateral quadratus lumborum (QL) block anatomy. IO: internal oblique; EO: external oblique; N: needle; QL: quadratus lumborum; TA: transverse abdominis.

A pillow was placed under the lumbar region in the supine position for quadratus lumborum 1 (QL1) or lateral quadratus lumborum block (QLB). After sterilizing the skin, 2 mL of 1% lidocaine was subcutaneously infiltrated to provide topical anesthesia. The same device was used to perform a long-axis in-plane ultrasound at the level of the anterior axillary line between the costal margin and the iliac crest. The transversus abdominis muscle (TAM), internal oblique muscle (IOM), and external oblique muscle (EOM) were the three abdominal anterolateral muscles that required localization. The quadratus lumborum muscle (QLM), characterized by a hypoechogenic region, can be located by moving the probe posterolaterally where the disappearance of the TAM can be witnessed in the anatomical axillary posterior line. Using the same needle type, 0.3 mL/kg of 0.5% ropivacaine was injected into the lateral terminal site of the transverse abdominis muscle through hydrodissection (Figure 2).

### 2.5. Patient Care after Block

Patients were instructed to assume a seated position 20–30 minutes after block administration. If VAS was >4 in this position, 1 *μ*g/kg of fentanyl was administered intravenously and repeated every 5 min if required. The total dose of fentanyl consumed until the appropriate time for spinal anesthesia was recorded. For postoperative analgesia, intravenous patient-controlled analgesia (IV-PCA) containing 40 mg of morphine in 40 mL of normal saline was administered. Each time the patient pushed the button, 0.5 mg of morphine was delivered (with a 15 min lockout time). If the VAS was >4 or higher within the first 24 h after surgery, 2 mg of intravenous morphine was administered as rescue therapy, and the total amount of morphine was also calculated.

The primary outcome was the analgesia duration (the time since the patient's first request for postoperative analgesia). Other variables included VAS scores at baseline (before the block procedure), in the recovery room (15 min after block performance), and 6, 12, and 24 h after surgery. In addition, the total dose of fentanyl for painless placement in the sitting position and the total amount of morphine administered in the first 24 h after surgery were evaluated. Moreover, blood pressure and heart rate were recorded at baseline and 15 minutes after the block procedure.

The patients, the block quality assessor, the anesthesia assistant responsible for intraoperative data collection, and the statistician were blinded to the block type. We utilized the Stoppa method as one of the standard surgical procedures for acetabular fractures.

### 2.6. Statistical Analysis

The chi-square test was used to evaluate categorical data, which were then expressed as frequency (percentage). The Kolmogorov–Smirnov test was utilized to demonstrate the normality of continuous data, and the data were expressed as the mean ± SD. The Mann–Whitney *U* test or independent-sample *t*-test was employed to compare continuous data between two study groups. Repeated-measures analysis of variance (ANOVA) was performed to analyze VAS at various times and block types (within-groups factor). Multiple comparisons (VAS) were corrected using the Bonferroni method. *P* values below 0.05 were considered statistically significant. SPSS software (v. 16.0) was utilized for data analysis.

## 3. Results

**Figure 3:**
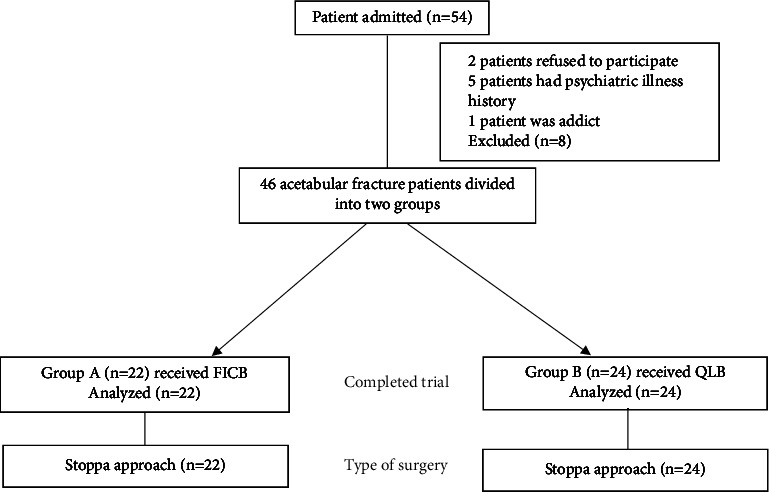
The study's flowchart. FICB: fascia iliaca compartment block; QLB: quadratus lumborum block.

This project enrolled 54 patients with acetabular fractures between August 2020 and March 2021. Eight patients were excluded from this study: two did not participate, five had a history of psychiatric illness, and one was addicted to multiple drugs. The remaining 46 subjects eventually completed the study and were analyzed. The remaining 46 patients were randomly divided into two groups (group FICB, *n* = 22; group QLB, *n* = 24) and underwent surgery using the Stoppa method (Figure 3). The patient demographics are shown in Table 1.

Our results indicated that both FICB and QLB led to significant reductions in blood pressure compared to baseline 20 min after administration (*P* < 0.001 and *P* = 0.019, respectively). In addition, 20 minutes after QLB, the heart rate was significantly lower than at baseline (*P* < 0.001), whereas there was no significant difference in this variable between the QLB and FICB groups (*P* = 0.89). A repeated-measures ANOVA with a Greenhouse–Geisser correction showed that the mean VAS scores significantly decreased compared to baseline in both FICB (*F* (3.37, 77.43) = 22.49, *P* < 0.001) and QLB groups (*F* (3.37, 77.43) = 22.49, *P* < 0.001). Bonferroni adjustment revealed that VAS scores decreased significantly in both groups compared to baseline, at recovery, and 6 h, 12 h, and 24 h following surgery (Tables 2 and 3). At any point during the trial, there was no significant difference between the two groups' VAS scores (Table 4). As shown in Table 5, total morphine requirements during the first 24 hours after surgery were significantly lower in the QLB group than in the FICB group. In contrast, total fentanyl consumption during spinal positioning was significantly higher in the QLB group than in the FICB group.

## 4. Discussion

The current study found no significant difference in VAS values between the two groups at any point during the study, and both FICB and QLB had a relatively similar postoperative analgesic profile after acetabular fracture surgery using the Stoppa method. Typically, each block offers an additional benefit. While the QLB group used significantly less morphine during the first 24 h after surgery than the FICB group, the FICB group had a lower cumulative fentanyl intake while positioned for spinal anesthesia. Nevertheless, in both methods, pain reduction occurred before surgery and during the positioning of the patient for spinal anesthesia, which is supposed to be due to the nerve blocks and fentanyl pretreatment.

FICB is already reported to provide perioperative analgesia after femoral neck fracture, total hip arthroplasty, and hip and knee surgery [6]. According to most existing studies and meta-analyses, FICB reduces pain intensity, the demand for opioids, and the rates of problems associated with their systemic use in these procedures [18–20]. Vergari et al. concur with our conclusion that FICB is a safe and effective option for postoperative analgesia following acetabular surgery [9]. As the lumbar plexus (LP) innervates the acetabular region, LP blocks provide analgesia in patients undergoing acetabular fracture surgery [9, 21]. The lumbar plexus comprises the obturator nerve (ON), lateral femoral cutaneous nerve, ilioinguinal nerve, iliohypogastric nerve, genitofemoral nerve, and femoral nerve (FN), as well as the lumbosacral trunk [22]. Theoretically, the possible mechanism of FICB block is blocking the femoral nerve, the lateral femoral cutaneous nerve, and the ON [9].

QLB is a relatively new regional block. Analgesic and opioid-sparing effects of QLB through all approaches (anterior, posterior, and lateral) have been demonstrated in several surgical procedures, including hip surgery [23], above-knee amputation [24], abdominal hernia repair [25], breast reconstruction [26], colostomy closure [27], radical nephrectomy [28], and total hip arthroplasty (THA) [15, 29, 30].

To our knowledge, no research has documented the analgesic efficacy of lateral QLB in acetabular fractures. Our results revealed that QLB decreased pain VAS scores and the need for opioids in the first postoperative 24 h. Nassar et al. found comparable outcomes, reporting that QLB can decrease the VAS score throughout spinal block positioning and increase postoperative motor power after THA [31]. Kukreja et al. observed that QLB could decrease pain intensity and the demand for analgesic medications 24 h following THA surgery [32]. However, using the same approach, Aoyama et al. were unable to detect sensory blockage of the lumbar nerves following transmuscular QLB [33].

As stated previously, systemic opioid administration has historically been used to treat pain after acetabular fracture procedures. This method has several disadvantages, including postoperative nausea, vomiting, oversedation, apnea, respiratory issues, and altered gastrointestinal function [4]. Consequently, utilizing analgesic techniques such as QLB that lessen the requirement for opioids can result in fewer side effects, early involvement in physical therapy, and quicker recovery and discharge [5, 7].

The precise mechanism of the analgesic effect of QLB remains unknown. Nonetheless, several potential mechanisms may be involved, including (a) medial distribution of the local anesthetic drug to the paravertebral spaces of the thoracolumbar region; (b) direct spread of local anesthetics to the lumbar plexus nerve roots and branches, such as the lateral femoral cutaneous, ilioinguinal, superior cluneal, and iliohypogastric nerves; inconsistent anesthetization of the femoral nerve, obturator nerve, and lumbar sympathetic trunk; and (c) the possibility of lumbar plexus block by spreading through the fascial layer between the psoas muscle [31].

## 5. Conclusion

In conclusion, our paper described the lateral QLB and FICB as effective analgesic techniques for acetabular fracture surgery utilizing the Stoppa approach, which exhibited a virtually identical analgesic profile. However, large-scale clinical studies must confirm these pilot clinical data to exclude local factors that could influence the final results.

## Figures and Tables

**Table 1:** Patients' demographics.

	FICB (*N* = 22)	QLB (*N* = 24)	*P* value
Age (years)	42.8 ± 10.9	41.5 ± 9.9	0.71
Sex (M/F)	18/4	14/10	0.087
Weight (kg)	72 ± 5.2	71.2 ± 7.8	0.72

Information is displayed as mean ± standard deviation. FICB, fascia iliaca block; QLB, quadratus lumborum block.

**Table 2:** VAS score changes at different times of the study compared to the baseline in the FICB group.

Variable	95% CI	*P* value
Recovery room VAS score	0.77–2.5	0.001
Spinal anesthesia positioning VAS score	0.71–2.56	0.001
VAS score before recovery delivery	0.72-0.72	1
Postsurgery VAS score (6 h)	0.21–1.97	0.007
Postsurgery VAS score (12 h)	0.16–2.16	0.15
Postsurgery VAS score (24 h)	0.32–2.58	0.005

**Table 3:** VAS score changes at different times of the study compared to the baseline in the QLB group.

variable	95% CI	*P* value
VAS score in recovery	1.38–4.12	0.001
Spinal anesthesia positioning VAS score	2.46–3.37	0.001
VAS score before recovery delivery	−0.1–71.5	0.26
Postsurgery VAS score (6 h)	0.94–3.22	0.001
Postsurgery VAS score (12 h)	1.95–4.05	0.001
Postsurgery vas score (24 h)	2.21–4.12	0.01

**Table 4:** Comparison of VAS score between two blocks.

	FICB (*N* = 22)	QLB (*N* = 24)	*P* value
Baseline VAS score (before blocks)	5.35 ± 0.93	5.75 ± 0.44	0.117
VAS score in recovery	3.27 ± 1.45	3.42 ± 2.18	0.80
Spinal anesthesia positioning VAS score	3.27 ± 1.24	3.25 ± 0.44	0.94
Postsurgery VAS score (6 h)	4.08 ± 1.58	3.82 ± 1.43	0.56
Postsurgery VAS score (12 h)	3.91 ± 1.48	3.17 ± 1.55	0.10
Postsurgery VAS score (24 h)	3.45 ± 1.33	3 ± 1.32	0.25

Information is displayed as mean ± standard deviation. FICB, fascia iliaca block; QLB, quadratus lumborum block; VAS, visual analog scale.

**Table 5:** Outcomes and block characteristics.

	FICB (*N* = 22)	QLB (*N* = 24)	*P* value
Fentanyl dosage for pain-free positioning in the seated position (mg)	105 ± 35.91	129.17 ± 38.78	0.039
Duration of analgesia (min)	281.36 ± 55.25	245.42 ± 78.03	0.081
The total amount of morphine taken over 24 h (mg)	16.7 ± 4.86	13 ± 5.24	0.02
Baseline heart rate (bpm)	80.63 ± 9.47	97.91 ± 16.76	<0.001
Heart rate (bpm) 20 min postblock	80.27 ± 14.49	92.18 ± 12.75	0.006
Baseline BP (mmHg)	130 ± 19.02	121.33 ± 12.03	0.069
BP (mmHg) 20 min postblock	114 ± 12.42	117.75 ± 11.97	0.30

Information is displayed as mean ± standard deviation. BP, blood pressure; FIB, fascia iliaca block; QLB, quadratus lumborum block; VAS, visual analog scale.

## Data Availability

The CONSORT and raw datasets used during the current study were uploaded as supplemental files alongside the manuscript submission. Also, they would be available from the corresponding author upon reasonable request.

## Additional Points

*Strengths and Limitations* The current study was the first to evaluate the potential analgesic effect of QLB in acetabular fracture surgeries using the Stoppa approach, providing a basis for future research in this field. However, this study has some limitations. First, we did not consider all the important parameters for evaluating the efficacy of enhanced recovery after surgery (ERAS), such as the time to first ambulation, length of hospitalization, and patient satisfaction. Second is the failure to evaluate the long-term side effects of the blocks, such as the development of chronic pain in the study groups. Third, the data from this study are insufficient to draw robust conclusions, and a larger sample size of randomized controlled trials is required to validate our results.
