# Supplementary material for: Quadratus Lumborum Block versus Fascia Iliaca Compartment Block for Acetabular Fracture Surgery by Stoppa Method: A Double-Blind, Randomized, Noninferiority Trial
Source: Pain Res Manag. 2024 Jan 5;2024:3720344. doi: 10.1155/2024/3720344 (PMC10787012; doi:10.1155/2024/3720344)
Supplement: Supplementary Materials — The CONSORT and raw datasets of this paper can be found in the supplementary material. [file 3720344.f1.zip › Raw data (1).docx]

| Groupe | Age | Weight | Gender | dose | drug | BPC1 | BPC2 | BPC3 | BPC4 | BPC5 | BPC6 | BPC7 | BPC8 | BPC9 | BPC10 | BPC11 | BPC12 | BPC13 | BPC14 | BPC15 | BPD1 | BPD2 | BPD3 | BPD4 | BPD5 | BPD6 | BPD7 | BPD8 | BPD9 | BPD10 | BPD11 | BPD12 | BPD13 | BPD14 | BPD15 | HR1 | HR2 | HR3 | HR4 | HR5 | HR6 | HR7 | HR8 | HR9 | HR10 | HR11 | HR12 | HR13 | HR14 | HR15 | Phentanyl | Analgesia | Morphin | VAS1 | VAS2 | VAS3 | VAS4 | VAS5 | VAS6 | VAS7 |
| --- | --- | --- | --- | --- | --- | --- | --- | --- | --- | --- | --- | --- | --- | --- | --- | --- | --- | --- | --- | --- | --- | --- | --- | --- | --- | --- | --- | --- | --- | --- | --- | --- | --- | --- | --- | --- | --- | --- | --- | --- | --- | --- | --- | --- | --- | --- | --- | --- | --- | --- | --- | --- | --- | --- | --- | --- | --- | --- | --- | --- |
| 1.00 | 46.00 | 70.00 | 1.00 | 20.00 | 20.00 | 140.00 | 120.00 | 112.00 | 107.00 | 120.00 | 112.00 | 109.00 | 103.00 | 123.00 | 129.00 | 134.00 | 130.00 | 110.00 | 110.00 | 101.00 | 80.00 | 80.00 | 95.00 | 88.00 | 80.00 | 63.00 | 62.00 | 70.00 | 77.00 | 77.00 | 77.00 | 76.00 | 60.00 | 70.00 | 60.00 | 85.00 | 80.00 | 110.00 | 100.00 | 100.00 | 104.00 | 108.00 | 115.00 | 113.00 | 120.00 | 132.00 | 108.00 | 117.00 | 110.00 | 107.00 | 0.00 | 1.00 | 10.00 | 3.00 | 1.00 | 1.00 | 3.00 | 1.00 | 2.00 | 2.00 |
| 1.00 | 46.00 | 75.00 | 1.00 | 25.00 | 20.00 | 130.00 | 125.00 | 120.00 | 110.00 | 100.00 | 85.00 | 78.00 | 80.00 | 81.00 | 90.00 | 88.00 | 80.00 | 100.00 | 102.00 | 110.00 | 70.00 | 75.00 | 80.00 | 75.00 | 80.00 | 65.00 | 50.00 | 40.00 | 65.00 | 60.00 | 65.00 | 60.00 | 60.00 | 70.00 | 70.00 | 80.00 | 84.00 | 78.00 | 80.00 | 87.00 | 86.00 | 80.00 | 86.00 | 86.00 | 88.00 | 80.00 | 80.00 | 82.00 | 78.00 | 84.00 | 100.00 | 3.00 | 25.00 | 6.00 | 5.00 | 2.00 | 6.00 | 6.00 | 7.00 | 6.00 |
| 1.00 | 45.00 | 80.00 | 1.00 | 25.00 | 20.00 | 140.00 | 130.00 | 140.00 | 130.00 | 135.00 | 90.00 | 94.00 | 100.00 | 106.00 | 115.00 | 110.00 | 100.00 | 110.00 | 110.00 | 100.00 | 90.00 | 90.00 | 80.00 | 76.00 | 78.00 | 60.00 | 60.00 | 60.00 | 70.00 | 65.00 | 70.00 | 60.00 | 70.00 | 60.00 | 60.00 | 82.00 | 80.00 | 83.00 | 80.00 | 78.00 | 70.00 | 70.00 | 70.00 | 78.00 | 76.00 | 70.00 | 68.00 | 75.00 | 78.00 | 76.00 | 150.00 | 1.00 | 20.00 | 5.00 | 4.00 | 4.00 | 4.00 | 5.00 | 4.00 | 4.00 |
| 1.00 | 46.00 | 75.00 | 1.00 | 20.00 | 4.00 | 150.00 | 130.00 | 130.00 | 120.00 | 120.00 | 90.00 | 90.00 | 90.00 | 100.00 | 109.00 | 110.00 | 115.00 | 115.00 | 120.00 | 125.00 | 80.00 | 70.00 | 70.00 | 70.00 | 50.00 | 50.00 | 60.00 | 60.00 | 60.00 | 70.00 | 90.00 | 75.00 | 80.00 | 70.00 | 70.00 | 78.00 | 77.00 | 70.00 | 72.00 | 80.00 | 78.00 | 83.00 | 83.00 | 78.00 | 79.00 | 80.00 | 85.00 | 84.00 | 86.00 | 80.00 | 50.00 | 1.00 | #NULL! | 6.00 | 3.00 | 4.00 | 4.00 | 5.00 | 6.00 | 4.00 |
| 1.00 | 22.00 | 80.00 | 1.00 | 25.00 | 20.00 | 120.00 | 118.00 | 112.00 | 100.00 | 110.00 | 110.00 | 80.00 | 97.00 | 82.00 | 84.00 | 96.00 | 90.00 | 88.00 | 100.00 | 100.00 | 80.00 | 70.00 | 70.00 | 60.00 | 60.00 | 70.00 | 60.00 | 62.00 | 50.00 | 50.00 | 60.00 | 50.00 | 60.00 | 60.00 | 56.00 | 80.00 | 110.00 | 107.00 | 105.00 | 95.00 | 96.00 | 90.00 | 90.00 | 90.00 | 90.00 | 90.00 | 84.00 | 92.00 | 90.00 | 86.00 | 100.00 | 4.00 | 12.00 | 4.00 | 3.00 | 3.00 | 4.00 | 3.00 | 4.00 | 4.00 |
| 1.00 | 46.00 | 75.00 | 1.00 | 25.00 | 20.00 | 130.00 | 117.00 | 130.00 | 119.00 | 120.00 | 110.00 | 102.00 | 90.00 | 92.00 | 90.00 | 92.00 | 90.00 | 110.00 | 102.00 | 110.00 | 70.00 | 80.00 | 70.00 | 80.00 | 80.00 | 70.00 | 50.00 | 60.00 | 70.00 | 60.00 | 56.00 | 60.00 | 70.00 | 70.00 | 70.00 | 70.00 | 60.00 | 60.00 | 50.00 | 50.00 | 56.00 | 61.00 | 60.00 | 62.00 | 60.00 | 58.00 | 57.00 | 66.00 | 70.00 | 70.00 | 100.00 | 4.00 | 12.00 | 2.00 | 1.00 | 1.00 | 4.00 | 2.00 | 2.00 | 1.00 |
| 1.00 | 45.00 | 70.00 | 1.00 | 20.00 | 20.00 | 120.00 | 122.00 | 130.00 | 112.00 | 110.00 | 115.00 | 110.00 | 111.00 | 110.00 | 110.00 | 110.00 | 100.00 | 107.00 | 110.00 | 110.00 | 70.00 | 70.00 | 70.00 | 75.00 | 70.00 | 76.00 | 60.00 | 75.00 | 60.00 | 60.00 | 70.00 | 60.00 | 70.00 | 70.00 | 70.00 | 90.00 | 92.00 | 90.00 | 80.00 | 82.00 | 76.00 | 78.00 | 76.00 | 70.00 | 70.00 | 72.00 | 72.00 | 74.00 | 74.00 | 74.00 | 150.00 | 3.00 | 20.00 | 8.00 | 4.00 | 6.00 | 6.00 | 5.00 | 4.00 | 4.00 |
| 1.00 | 42.00 | 78.00 | 1.00 | 25.00 | 20.00 | 100.00 | 105.00 | 108.00 | 96.00 | 109.00 | 110.00 | 100.00 | 96.00 | 85.00 | 88.00 | 88.00 | 83.00 | 85.00 | 90.00 | 86.00 | 60.00 | 60.00 | 65.00 | 70.00 | 50.00 | 70.00 | 60.00 | 70.00 | 53.00 | 70.00 | 60.00 | 56.00 | 56.00 | 60.00 | 60.00 | 72.00 | 76.00 | 73.00 | 70.00 | 70.00 | 75.00 | 75.00 | 80.00 | 86.00 | 74.00 | 75.00 | 78.00 | 80.00 | 80.00 | 80.00 | 150.00 | 1.00 | 12.00 | 3.00 | 4.00 | 4.00 | 4.00 | 4.00 | 4.00 | 4.00 |
| 1.00 | 52.00 | 70.00 | 1.00 | 20.00 | 24.00 | 120.00 | 110.00 | 103.00 | 108.00 | 101.00 | 80.00 | 93.00 | 110.00 | 98.00 | 87.00 | 110.00 | 115.00 | 120.00 | 110.00 | 110.00 | 70.00 | 70.00 | 70.00 | 70.00 | 60.00 | 60.00 | 60.00 | 70.00 | 70.00 | 60.00 | 70.00 | 70.00 | 70.00 | 70.00 | 70.00 | 85.00 | 88.00 | 80.00 | 80.00 | 90.00 | 90.00 | 84.00 | 77.00 | 85.00 | 87.00 | 90.00 | 87.00 | 70.00 | 80.00 | 80.00 | 100.00 | 5.00 | #NULL! | 5.00 | 3.00 | 3.00 | 5.00 | 3.00 | 3.00 | 3.00 |
| 1.00 | 48.00 | 70.00 | 1.00 | 20.00 | 20.00 | 140.00 | 120.00 | 112.00 | 107.00 | 120.00 | 112.00 | 109.00 | 103.00 | 123.00 | 129.00 | 134.00 | 130.00 | 110.00 | 110.00 | 101.00 | 80.00 | 80.00 | 95.00 | 88.00 | 80.00 | 63.00 | 62.00 | 70.00 | 77.00 | 77.00 | 77.00 | 76.00 | 60.00 | 70.00 | 60.00 | 85.00 | 80.00 | 110.00 | 100.00 | 100.00 | 104.00 | 108.00 | 115.00 | 113.00 | 120.00 | 132.00 | 108.00 | 117.00 | 110.00 | 107.00 | 0.00 | 1.00 | 10.00 | 3.00 | 1.00 | 1.00 | 3.00 | 1.00 | 2.00 | 2.00 |
| 1.00 | 46.00 | 75.00 | 1.00 | 25.00 | 20.00 | 130.00 | 125.00 | 120.00 | 110.00 | 100.00 | 85.00 | 78.00 | 80.00 | 81.00 | 90.00 | 88.00 | 80.00 | 100.00 | 102.00 | 110.00 | 70.00 | 75.00 | 80.00 | 75.00 | 80.00 | 65.00 | 50.00 | 40.00 | 65.00 | 60.00 | 65.00 | 60.00 | 60.00 | 70.00 | 70.00 | 80.00 | 84.00 | 78.00 | 80.00 | 87.00 | 86.00 | 80.00 | 86.00 | 86.00 | 88.00 | 80.00 | 80.00 | 82.00 | 78.00 | 84.00 | 100.00 | 3.00 | 25.00 | 6.00 | 5.00 | 2.00 | 6.00 | 6.00 | 7.00 | 6.00 |
| 1.00 | 45.00 | 80.00 | 1.00 | 25.00 | 20.00 | 140.00 | 130.00 | 140.00 | 130.00 | 135.00 | 90.00 | 94.00 | 100.00 | 106.00 | 115.00 | 110.00 | 100.00 | 110.00 | 110.00 | 100.00 | 90.00 | 90.00 | 80.00 | 76.00 | 78.00 | 60.00 | 60.00 | 60.00 | 70.00 | 65.00 | 70.00 | 60.00 | 70.00 | 60.00 | 60.00 | 82.00 | 80.00 | 83.00 | 80.00 | 78.00 | 70.00 | 70.00 | 70.00 | 78.00 | 76.00 | 70.00 | 68.00 | 75.00 | 78.00 | 76.00 | 150.00 | 1.00 | 20.00 | 5.00 | 4.00 | 4.00 | 4.00 | 5.00 | 4.00 | 4.00 |
| 1.00 | 46.00 | 75.00 | 1.00 | 20.00 | 4.00 | 150.00 | 130.00 | 130.00 | 120.00 | 120.00 | 90.00 | 90.00 | 90.00 | 100.00 | 109.00 | 110.00 | 115.00 | 115.00 | 120.00 | 125.00 | 80.00 | 70.00 | 70.00 | 70.00 | 50.00 | 50.00 | 60.00 | 60.00 | 60.00 | 70.00 | 90.00 | 75.00 | 80.00 | 70.00 | 70.00 | 78.00 | 77.00 | 70.00 | 72.00 | 80.00 | 78.00 | 83.00 | 83.00 | 78.00 | 79.00 | 80.00 | 85.00 | 84.00 | 86.00 | 80.00 | 50.00 | 1.00 | #NULL! | 6.00 | 3.00 | 4.00 | 4.00 | 5.00 | 6.00 | 4.00 |
| 1.00 | 22.00 | 80.00 | 1.00 | 25.00 | 20.00 | 120.00 | 118.00 | 112.00 | 100.00 | 110.00 | 110.00 | 80.00 | 97.00 | 82.00 | 84.00 | 96.00 | 90.00 | 88.00 | 100.00 | 100.00 | 80.00 | 70.00 | 70.00 | 60.00 | 60.00 | 70.00 | 60.00 | 62.00 | 50.00 | 50.00 | 60.00 | 50.00 | 60.00 | 60.00 | 56.00 | 80.00 | 110.00 | 107.00 | 105.00 | 95.00 | 96.00 | 90.00 | 90.00 | 90.00 | 90.00 | 90.00 | 84.00 | 92.00 | 90.00 | 86.00 | 100.00 | 4.00 | 12.00 | 4.00 | 3.00 | 3.00 | 4.00 | 3.00 | 4.00 | 4.00 |
| 1.00 | 46.00 | 75.00 | 1.00 | 25.00 | 20.00 | 130.00 | 117.00 | 130.00 | 119.00 | 120.00 | 110.00 | 102.00 | 90.00 | 92.00 | 90.00 | 92.00 | 90.00 | 110.00 | 102.00 | 110.00 | 70.00 | 80.00 | 70.00 | 80.00 | 80.00 | 70.00 | 50.00 | 60.00 | 70.00 | 60.00 | 56.00 | 60.00 | 70.00 | 70.00 | 70.00 | 70.00 | 60.00 | 60.00 | 50.00 | 50.00 | 56.00 | 61.00 | 60.00 | 62.00 | 60.00 | 58.00 | 57.00 | 66.00 | 70.00 | 70.00 | 100.00 | 4.00 | 12.00 | 2.00 | 1.00 | 1.00 | 4.00 | 2.00 | 2.00 | 1.00 |
| 1.00 | 45.00 | 70.00 | 1.00 | 20.00 | 20.00 | 120.00 | 122.00 | 130.00 | 112.00 | 110.00 | 115.00 | 110.00 | 111.00 | 110.00 | 110.00 | 110.00 | 100.00 | 107.00 | 110.00 | 110.00 | 70.00 | 70.00 | 70.00 | 75.00 | 70.00 | 76.00 | 60.00 | 75.00 | 60.00 | 60.00 | 70.00 | 60.00 | 70.00 | 70.00 | 70.00 | 90.00 | 92.00 | 90.00 | 80.00 | 82.00 | 76.00 | 78.00 | 76.00 | 70.00 | 70.00 | 72.00 | 72.00 | 74.00 | 74.00 | 74.00 | 150.00 | 3.00 | 20.00 | 8.00 | 4.00 | 6.00 | 6.00 | 5.00 | 4.00 | 4.00 |
| 1.00 | 42.00 | 78.00 | 1.00 | 25.00 | 20.00 | 100.00 | 105.00 | 108.00 | 96.00 | 109.00 | 110.00 | 100.00 | 96.00 | 85.00 | 88.00 | 88.00 | 83.00 | 85.00 | 90.00 | 86.00 | 60.00 | 60.00 | 65.00 | 70.00 | 50.00 | 70.00 | 60.00 | 70.00 | 53.00 | 70.00 | 60.00 | 56.00 | 56.00 | 60.00 | 60.00 | 72.00 | 76.00 | 73.00 | 70.00 | 70.00 | 75.00 | 75.00 | 80.00 | 86.00 | 74.00 | 75.00 | 78.00 | 80.00 | 80.00 | 80.00 | 150.00 | 1.00 | 12.00 | 3.00 | 4.00 | 4.00 | 4.00 | 4.00 | 4.00 | 4.00 |
| 1.00 | 45.00 | 70.00 | 1.00 | 20.00 | 24.00 | 120.00 | 110.00 | 103.00 | 108.00 | 101.00 | 80.00 | 93.00 | 110.00 | 98.00 | 87.00 | 110.00 | 115.00 | 120.00 | 110.00 | 110.00 | 70.00 | 70.00 | 70.00 | 70.00 | 60.00 | 60.00 | 60.00 | 70.00 | 70.00 | 60.00 | 70.00 | 70.00 | 70.00 | 70.00 | 70.00 | 85.00 | 88.00 | 80.00 | 80.00 | 90.00 | 90.00 | 84.00 | 77.00 | 85.00 | 87.00 | 90.00 | 87.00 | 70.00 | 80.00 | 80.00 | 100.00 | 5.00 | #NULL! | 5.00 | 3.00 | 3.00 | 5.00 | 3.00 | 3.00 | 3.00 |
| 2.00 | 60.00 | 75.00 | 1.00 | 20.00 | 20.00 | 110.00 | 120.00 | 116.00 | 110.00 | 120.00 | 100.00 | 105.00 | 102.00 | 100.00 | 109.00 | 110.00 | 111.00 | 117.00 | 106.00 | 105.00 | 70.00 | 78.00 | 57.00 | 90.00 | 96.00 | 60.00 | 56.00 | 63.00 | 56.00 | 59.00 | 60.00 | 70.00 | 60.00 | 70.00 | 65.00 | 90.00 | 86.00 | 80.00 | 82.00 | 75.00 | 77.00 | 70.00 | 71.00 | 72.00 | 70.00 | 73.00 | 74.00 | 75.00 | 76.00 | 78.00 | 150.00 | 1.00 | 12.00 | 5.00 | 3.00 | 6.00 | 6.00 | 4.00 | 2.00 | 2.00 |
| 2.00 | 38.00 | 80.00 | 1.00 | 25.00 | 20.00 | 110.00 | 100.00 | 126.00 | 116.00 | 115.00 | 100.00 | 96.00 | 97.00 | 90.00 | 96.00 | 93.00 | 90.00 | 90.00 | 90.00 | #NULL! | 70.00 | 60.00 | 72.00 | 69.00 | 70.00 | 62.00 | 50.00 | 53.00 | 50.00 | 60.00 | 70.00 | 59.00 | 60.00 | 63.00 | #NULL! | 96.00 | 98.00 | 97.00 | 90.00 | 92.00 | 80.00 | 82.00 | 76.00 | 77.00 | 76.00 | 79.00 | 76.00 | 74.00 | 76.00 | 75.00 | 150.00 | 2.00 | 16.00 | 6.00 | 3.00 | 1.00 | 7.00 | 2.00 | 3.00 | 1.00 |
| 2.00 | 52.00 | 76.00 | 1.00 | 20.00 | 20.00 | 126.00 | 126.00 | 110.00 | 116.00 | 110.00 | 106.00 | 100.00 | 102.00 | 100.00 | 96.00 | 99.00 | 96.00 | 107.00 | 100.00 | 105.00 | 70.00 | 76.00 | 70.00 | 60.00 | 80.00 | 70.00 | 60.00 | 70.00 | 62.00 | 70.00 | 62.00 | 70.00 | 70.00 | 56.00 | 56.00 | 110.00 | 108.00 | 108.00 | 106.00 | 100.00 | 96.00 | 100.00 | 90.00 | 86.00 | 80.00 | 82.00 | 82.00 | 80.00 | 86.00 | 90.00 | 100.00 | 1.00 | 19.00 | 5.00 | 3.00 | 4.00 | 5.00 | 2.00 | 1.00 | 2.00 |
| 2.00 | 28.00 | 72.00 | 1.00 | 20.00 | 20.00 | 120.00 | 122.00 | 120.00 | 130.00 | 110.00 | 135.00 | 117.00 | 120.00 | 130.00 | 120.00 | 117.00 | 120.00 | 130.00 | 128.00 | 120.00 | 75.00 | 76.00 | 80.00 | 78.00 | 60.00 | 80.00 | 76.00 | 80.00 | 85.00 | 80.00 | 70.00 | 75.00 | 80.00 | 78.00 | 76.00 | 80.00 | 75.00 | 80.00 | 82.00 | 78.00 | 85.00 | 90.00 | 95.00 | 94.00 | 98.00 | 95.00 | 94.00 | 98.00 | 96.00 | 96.00 | 150.00 | 1.00 | #NULL! | 6.00 | 3.00 | 3.00 | 6.00 | 2.00 | 5.00 | 5.00 |
| 2.00 | 30.00 | 82.00 | 1.00 | 20.00 | 24.00 | 110.00 | 119.00 | 110.00 | 112.00 | 126.00 | 127.00 | 117.00 | 110.00 | 116.00 | 114.00 | 116.00 | 116.00 | 117.00 | 120.00 | 110.00 | 70.00 | 76.00 | 70.00 | 70.00 | 70.00 | 76.00 | 70.00 | 76.00 | 72.00 | 76.00 | 70.00 | 70.00 | 69.00 | 70.00 | 70.00 | 90.00 | 92.00 | 90.00 | 86.00 | 86.00 | 80.00 | 86.00 | 90.00 | 92.00 | 90.00 | 89.00 | 89.00 | 88.00 | 88.00 | 90.00 | 200.00 | 6.00 | #NULL! | 8.00 | 4.00 | 8.00 | 8.00 | 4.00 | 4.00 | 3.00 |
| 2.00 | 45.00 | 60.00 | 1.00 | 18.00 | 20.00 | 140.00 | 139.00 | 130.00 | 130.00 | 130.00 | 130.00 | 132.00 | 128.00 | 120.00 | 110.00 | 112.00 | 110.00 | 120.00 | 128.00 | 129.00 | 90.00 | 87.00 | 80.00 | 80.00 | 70.00 | 80.00 | 76.00 | 70.00 | 70.00 | 70.00 | 72.00 | 70.00 | 80.00 | 90.00 | 89.00 | #NULL! | #NULL! | #NULL! | #NULL! | #NULL! | #NULL! | #NULL! | #NULL! | #NULL! | #NULL! | #NULL! | #NULL! | #NULL! | #NULL! | #NULL! | 100.00 | 6.00 | 20.00 | 8.00 | 4.00 | 6.00 | 6.00 | 5.00 | 4.00 | 5.00 |
| 2.00 | 32.00 | 72.00 | 1.00 | 20.00 | 20.00 | 130.00 | 125.00 | 120.00 | 120.00 | 125.00 | 110.00 | 108.00 | 100.00 | 101.00 | 100.00 | 96.00 | 99.00 | 90.00 | 92.00 | 90.00 | 70.00 | 70.00 | 70.00 | 66.00 | 67.00 | 60.00 | 60.00 | 65.00 | 68.00 | 60.00 | 50.00 | 50.00 | 60.00 | 56.00 | 60.00 | 100.00 | 98.00 | 98.00 | 94.00 | 90.00 | 90.00 | 92.00 | 85.00 | 86.00 | 80.00 | 80.00 | 78.00 | 80.00 | 76.00 | 76.00 | 150.00 | 1.00 | 8.00 | 7.00 | 4.00 | 2.00 | 7.00 | 5.00 | 5.00 | 3.00 |
| 2.00 | 36.00 | 73.00 | 1.00 | 20.00 | 29.00 | 140.00 | 130.00 | 125.00 | 130.00 | 130.00 | 125.00 | 120.00 | 110.00 | 105.00 | 110.00 | 112.00 | 110.00 | 110.00 | 100.00 | 100.00 | 80.00 | 70.00 | 70.00 | 76.00 | 70.00 | 65.00 | 60.00 | 65.00 | 60.00 | 65.00 | 66.00 | 70.00 | 60.00 | 60.00 | 60.00 | 80.00 | 75.00 | 76.00 | 70.00 | 76.00 | 70.00 | 65.00 | 66.00 | 65.00 | 65.00 | 70.00 | 70.00 | 70.00 | 70.00 | 70.00 | 250.00 | 1.00 | 15.00 | 6.00 | 3.00 | 6.00 | 6.00 | 1.00 | 3.00 | 4.00 |
| 2.00 | 60.00 | 75.00 | 1.00 | 20.00 | 20.00 | 110.00 | 120.00 | 116.00 | 110.00 | 120.00 | 100.00 | 105.00 | 102.00 | 100.00 | 109.00 | 110.00 | 111.00 | 117.00 | 106.00 | 105.00 | 70.00 | 78.00 | 57.00 | 90.00 | 96.00 | 60.00 | 56.00 | 63.00 | 56.00 | 59.00 | 60.00 | 70.00 | 60.00 | 70.00 | 65.00 | 90.00 | 86.00 | 80.00 | 82.00 | 75.00 | 77.00 | 70.00 | 71.00 | 72.00 | 70.00 | 73.00 | 74.00 | 75.00 | 76.00 | 78.00 | 150.00 | 1.00 | 12.00 | 5.00 | 3.00 | 6.00 | 6.00 | 4.00 | 2.00 | 2.00 |
| 2.00 | 41.00 | 80.00 | 1.00 | 25.00 | 20.00 | 110.00 | 100.00 | 126.00 | 116.00 | 115.00 | 100.00 | 96.00 | 97.00 | 90.00 | 96.00 | 93.00 | 90.00 | 90.00 | 90.00 | #NULL! | 70.00 | 60.00 | 72.00 | 69.00 | 70.00 | 62.00 | 50.00 | 53.00 | 50.00 | 60.00 | 70.00 | 59.00 | 60.00 | 63.00 | #NULL! | 96.00 | 98.00 | 97.00 | 90.00 | 92.00 | 80.00 | 82.00 | 76.00 | 77.00 | 76.00 | 79.00 | 76.00 | 74.00 | 76.00 | 75.00 | 150.00 | 2.00 | 16.00 | 6.00 | 3.00 | 1.00 | 7.00 | 2.00 | 3.00 | 1.00 |
| 2.00 | 52.00 | 76.00 | 1.00 | 20.00 | 20.00 | 126.00 | 126.00 | 110.00 | 116.00 | 110.00 | 106.00 | 100.00 | 102.00 | 100.00 | 96.00 | 99.00 | 96.00 | 107.00 | 100.00 | 105.00 | 70.00 | 76.00 | 70.00 | 60.00 | 80.00 | 70.00 | 60.00 | 70.00 | 62.00 | 70.00 | 62.00 | 70.00 | 70.00 | 56.00 | 56.00 | 110.00 | 108.00 | 108.00 | 106.00 | 100.00 | 96.00 | 100.00 | 90.00 | 86.00 | 80.00 | 82.00 | 82.00 | 80.00 | 86.00 | 90.00 | 100.00 | 1.00 | 19.00 | 5.00 | 3.00 | 4.00 | 5.00 | 2.00 | 1.00 | 2.00 |
| 2.00 | 37.00 | 72.00 | 1.00 | 20.00 | 20.00 | 120.00 | 122.00 | 120.00 | 130.00 | 110.00 | 135.00 | 117.00 | 120.00 | 130.00 | 120.00 | 117.00 | 120.00 | 130.00 | 128.00 | 120.00 | 75.00 | 76.00 | 80.00 | 78.00 | 60.00 | 80.00 | 76.00 | 80.00 | 85.00 | 80.00 | 70.00 | 75.00 | 80.00 | 78.00 | 76.00 | 80.00 | 75.00 | 80.00 | 82.00 | 78.00 | 85.00 | 90.00 | 95.00 | 94.00 | 98.00 | 95.00 | 94.00 | 98.00 | 96.00 | 96.00 | 150.00 | 1.00 | #NULL! | 6.00 | 3.00 | 3.00 | 6.00 | 2.00 | 5.00 | 5.00 |
| 2.00 | 30.00 | 82.00 | 1.00 | 20.00 | 24.00 | 110.00 | 119.00 | 110.00 | 112.00 | 126.00 | 127.00 | 117.00 | 110.00 | 116.00 | 114.00 | 116.00 | 116.00 | 117.00 | 120.00 | 110.00 | 70.00 | 76.00 | 70.00 | 70.00 | 70.00 | 76.00 | 70.00 | 76.00 | 72.00 | 76.00 | 70.00 | 70.00 | 69.00 | 70.00 | 70.00 | 90.00 | 92.00 | 90.00 | 86.00 | 86.00 | 80.00 | 86.00 | 90.00 | 92.00 | 90.00 | 89.00 | 89.00 | 88.00 | 88.00 | 90.00 | 200.00 | 6.00 | #NULL! | 8.00 | 4.00 | 8.00 | 8.00 | 4.00 | 4.00 | 3.00 |
| 2.00 | 45.00 | 60.00 | 1.00 | 18.00 | 20.00 | 140.00 | 139.00 | 130.00 | 130.00 | 130.00 | 130.00 | 132.00 | 128.00 | 120.00 | 110.00 | 112.00 | 110.00 | 120.00 | 128.00 | 129.00 | 90.00 | 87.00 | 80.00 | 80.00 | 70.00 | 80.00 | 76.00 | 70.00 | 70.00 | 70.00 | 72.00 | 70.00 | 80.00 | 90.00 | 89.00 | #NULL! | #NULL! | #NULL! | #NULL! | #NULL! | #NULL! | #NULL! | #NULL! | #NULL! | #NULL! | #NULL! | #NULL! | #NULL! | #NULL! | #NULL! | 100.00 | 6.00 | 20.00 | 8.00 | 4.00 | 6.00 | 6.00 | 5.00 | 4.00 | 5.00 |
| 2.00 | 32.00 | 72.00 | 1.00 | 20.00 | 20.00 | 130.00 | 125.00 | 120.00 | 120.00 | 125.00 | 110.00 | 108.00 | 100.00 | 101.00 | 100.00 | 96.00 | 99.00 | 90.00 | 92.00 | 90.00 | 70.00 | 70.00 | 70.00 | 66.00 | 67.00 | 60.00 | 60.00 | 65.00 | 68.00 | 60.00 | 50.00 | 50.00 | 60.00 | 56.00 | 60.00 | 100.00 | 98.00 | 98.00 | 94.00 | 90.00 | 90.00 | 92.00 | 85.00 | 86.00 | 80.00 | 80.00 | 78.00 | 80.00 | 76.00 | 76.00 | 150.00 | 1.00 | 8.00 | 7.00 | 4.00 | 2.00 | 7.00 | 5.00 | 5.00 | 3.00 |
| 2.00 | 36.00 | 73.00 | 1.00 | 20.00 | 29.00 | 140.00 | 130.00 | 125.00 | 130.00 | 130.00 | 125.00 | 120.00 | 110.00 | 105.00 | 110.00 | 112.00 | 110.00 | 110.00 | 100.00 | 100.00 | 80.00 | 70.00 | 70.00 | 76.00 | 70.00 | 65.00 | 60.00 | 65.00 | 60.00 | 65.00 | 66.00 | 70.00 | 60.00 | 60.00 | 60.00 | 80.00 | 75.00 | 76.00 | 70.00 | 76.00 | 70.00 | 65.00 | 66.00 | 65.00 | 65.00 | 70.00 | 70.00 | 70.00 | 70.00 | 70.00 | 250.00 | 1.00 | 15.00 | 6.00 | 3.00 | 6.00 | 6.00 | 1.00 | 3.00 | 4.00 |
| 1.00 | 42.00 | 60.00 | 2.00 | 15.00 | 20.00 | 110.00 | 109.00 | 110.00 | 112.00 | 120.00 | 100.00 | 90.00 | 89.00 | 88.00 | 90.00 | 92.00 | 88.00 | 90.00 | 91.00 | 93.00 | 70.00 | 71.00 | 70.00 | 72.00 | 80.00 | 70.00 | 62.00 | 60.00 | 59.00 | 60.00 | 57.00 | 60.00 | 56.00 | 70.00 | 50.00 | 100.00 | 98.00 | 90.00 | 90.00 | 92.00 | 86.00 | 80.00 | 82.00 | 80.00 | 78.00 | 80.00 | 76.00 | 78.00 | 76.00 | 76.00 | 100.00 | 1.00 | 10.00 | 6.00 | 3.00 | 4.00 | 4.00 | 6.00 | 3.00 | 2.00 |
| 1.00 | 45.00 | 75.00 | 2.00 | 20.00 | 20.00 | 140.00 | 150.00 | 150.00 | 140.00 | 120.00 | 120.00 | 130.00 | 130.00 | 130.00 | 110.00 | 120.00 | 120.00 | 110.00 | 110.00 | 120.00 | 60.00 | 80.00 | 70.00 | 90.00 | 80.00 | 65.00 | 80.00 | 80.00 | 90.00 | 70.00 | 70.00 | 70.00 | 70.00 | 75.00 | 70.00 | 65.00 | 70.00 | 60.00 | 76.00 | 70.00 | 70.00 | 76.00 | 80.00 | 80.00 | 80.00 | 75.00 | 65.00 | 66.00 | 66.00 | 68.00 | 50.00 | 3.00 | 16.00 | 6.00 | 4.00 | 4.00 | 6.00 | 4.00 | 4.00 | 4.00 |
| 1.00 | 42.00 | 60.00 | 2.00 | 15.00 | 20.00 | 110.00 | 109.00 | 110.00 | 112.00 | 120.00 | 100.00 | 90.00 | 89.00 | 88.00 | 90.00 | 92.00 | 88.00 | 90.00 | 91.00 | 93.00 | 70.00 | 71.00 | 70.00 | 72.00 | 80.00 | 70.00 | 62.00 | 60.00 | 59.00 | 60.00 | 57.00 | 60.00 | 56.00 | 70.00 | 50.00 | 100.00 | 98.00 | 90.00 | 90.00 | 92.00 | 86.00 | 80.00 | 82.00 | 80.00 | 78.00 | 80.00 | 76.00 | 78.00 | 76.00 | 76.00 | 100.00 | 1.00 | 10.00 | 6.00 | 3.00 | 4.00 | 4.00 | 6.00 | 3.00 | 2.00 |
| 1.00 | 45.00 | 75.00 | 2.00 | 20.00 | 20.00 | 140.00 | 150.00 | 150.00 | 140.00 | 120.00 | 120.00 | 130.00 | 130.00 | 130.00 | 110.00 | 120.00 | 120.00 | 110.00 | 110.00 | 120.00 | 60.00 | 80.00 | 70.00 | 90.00 | 80.00 | 65.00 | 80.00 | 80.00 | 90.00 | 70.00 | 70.00 | 70.00 | 70.00 | 75.00 | 70.00 | 65.00 | 70.00 | 60.00 | 76.00 | 70.00 | 70.00 | 76.00 | 80.00 | 80.00 | 80.00 | 75.00 | 65.00 | 66.00 | 66.00 | 68.00 | 50.00 | 3.00 | 16.00 | 6.00 | 4.00 | 4.00 | 6.00 | 4.00 | 4.00 | 4.00 |
| 2.00 | 34.00 | 68.00 | 2.00 | 15.00 | 20.00 | 115.00 | 110.00 | 106.00 | 100.00 | 90.00 | 92.00 | 94.00 | 88.00 | 89.00 | 80.00 | 82.00 | 80.00 | 88.00 | 100.00 | 100.00 | 56.00 | 70.00 | 60.00 | 70.00 | 60.00 | 56.00 | 60.00 | 56.00 | 70.00 | 60.00 | 54.00 | 50.00 | 62.00 | 60.00 | 60.00 | 110.00 | 112.00 | 104.00 | 100.00 | 100.00 | 96.00 | 94.00 | 90.00 | 90.00 | 92.00 | 94.00 | 90.00 | 92.00 | 90.00 | 100.00 | 100.00 | 2.00 | 10.00 | 6.00 | 3.00 | 2.00 | 4.00 | 3.00 | 1.00 | 3.00 |
| 2.00 | 40.00 | 68.00 | 2.00 | 20.00 | 20.00 | 135.00 | 130.00 | 125.00 | 127.00 | 126.00 | 130.00 | 126.00 | 125.00 | 125.00 | 124.00 | 124.00 | 125.00 | 116.00 | 110.00 | 110.00 | 90.00 | 90.00 | 85.00 | 90.00 | 90.00 | 80.00 | 80.00 | 50.00 | 85.00 | 83.00 | 80.00 | 80.00 | 77.00 | 70.00 | 70.00 | 115.00 | 108.00 | 105.00 | 100.00 | 100.00 | 97.00 | 101.00 | 97.00 | 97.00 | 93.00 | 98.00 | 96.00 | 95.00 | 103.00 | 103.00 | 150.00 | 2.00 | 10.00 | 6.00 | 3.00 | 1.00 | 4.00 | 7.00 | 1.00 | 1.00 |
| 2.00 | 52.00 | 64.00 | 2.00 | 18.00 | 4.00 | 140.00 | 145.00 | 140.00 | 142.00 | 140.00 | 130.00 | 125.00 | 126.00 | 110.00 | 105.00 | 106.00 | 107.00 | 100.00 | 103.00 | 105.00 | 90.00 | 93.00 | 90.00 | 91.00 | 85.00 | 70.00 | 71.00 | 70.00 | 64.00 | 64.00 | 70.00 | 67.00 | 61.00 | 70.00 | 66.00 | 60.00 | 66.00 | 66.00 | 64.00 | 70.00 | 64.00 | 60.00 | 60.00 | 62.00 | 60.00 | 62.00 | 62.00 | 66.00 | 62.00 | 63.00 | 50.00 | 1.00 | 6.00 | 5.00 | 3.00 | 2.00 | 3.00 | 4.00 | 5.00 | 4.00 |
| 2.00 | 33.00 | 65.00 | 2.00 | 15.00 | 4.00 | 110.00 | 112.00 | 110.00 | 106.00 | 110.00 | 100.00 | 105.00 | 100.00 | 98.00 | 90.00 | 90.00 | 96.00 | 95.00 | 94.00 | 96.00 | 60.00 | 70.00 | 70.00 | 69.00 | 70.00 | 60.00 | 67.00 | 68.00 | 70.00 | 60.00 | 60.00 | 70.00 | 65.00 | 60.00 | 66.00 | 116.00 | 118.00 | 110.00 | 108.00 | 110.00 | 100.00 | 98.00 | 90.00 | 86.00 | 80.00 | 80.00 | 78.00 | 79.00 | 78.00 | 76.00 | 100.00 | 1.00 | 8.00 | 6.00 | 3.00 | 2.00 | 6.00 | 5.00 | 4.00 | 4.00 |
| 2.00 | 35.00 | 62.00 | 2.00 | 12.00 | 4.00 | 110.00 | 115.00 | 105.00 | 110.00 | 115.00 | 113.00 | 103.00 | 103.00 | 100.00 | 101.00 | 97.00 | 97.00 | 100.00 | 98.00 | 100.00 | 70.00 | 70.00 | 67.00 | 72.00 | 70.00 | 60.00 | 60.00 | 56.00 | 60.00 | 61.00 | 58.00 | 58.00 | 60.00 | 60.00 | 70.00 | 110.00 | 108.00 | 100.00 | 102.00 | 110.00 | 100.00 | 96.00 | 97.00 | 90.00 | 89.00 | 85.00 | 80.00 | 80.00 | 81.00 | 80.00 | 150.00 | 6.00 | 20.00 | 6.00 | 3.00 | 4.00 | 4.00 | 6.00 | 3.00 | 3.00 |
| 2.00 | 42.00 | 82.00 | 2.00 | 15.00 | 20.00 | 115.00 | 110.00 | 106.00 | 100.00 | 90.00 | 92.00 | 94.00 | 88.00 | 89.00 | 80.00 | 82.00 | 80.00 | 88.00 | 100.00 | 100.00 | 56.00 | 70.00 | 60.00 | 70.00 | 60.00 | 56.00 | 60.00 | 56.00 | 70.00 | 60.00 | 54.00 | 50.00 | 62.00 | 60.00 | 60.00 | 110.00 | 112.00 | 104.00 | 100.00 | 100.00 | 96.00 | 94.00 | 90.00 | 90.00 | 92.00 | 94.00 | 90.00 | 92.00 | 90.00 | 100.00 | 100.00 | 2.00 | 10.00 | 6.00 | 3.00 | 2.00 | 4.00 | 3.00 | 1.00 | 3.00 |
| 2.00 | 40.00 | 68.00 | 2.00 | 20.00 | 20.00 | 135.00 | 130.00 | 125.00 | 127.00 | 126.00 | 130.00 | 126.00 | 125.00 | 125.00 | 124.00 | 124.00 | 125.00 | 116.00 | 110.00 | 110.00 | 90.00 | 90.00 | 85.00 | 90.00 | 90.00 | 80.00 | 80.00 | 50.00 | 85.00 | 83.00 | 80.00 | 80.00 | 77.00 | 70.00 | 70.00 | 115.00 | 108.00 | 105.00 | 100.00 | 100.00 | 97.00 | 101.00 | 97.00 | 97.00 | 93.00 | 98.00 | 96.00 | 95.00 | 103.00 | 103.00 | 150.00 | 2.00 | 10.00 | 6.00 | 3.00 | 1.00 | 4.00 | 7.00 | 1.00 | 1.00 |
| 2.00 | 52.00 | 64.00 | 2.00 | 18.00 | 4.00 | 140.00 | 145.00 | 140.00 | 142.00 | 140.00 | 130.00 | 125.00 | 126.00 | 110.00 | 105.00 | 106.00 | 107.00 | 100.00 | 103.00 | 105.00 | 90.00 | 93.00 | 90.00 | 91.00 | 85.00 | 70.00 | 71.00 | 70.00 | 64.00 | 64.00 | 70.00 | 67.00 | 61.00 | 70.00 | 66.00 | 60.00 | 66.00 | 66.00 | 64.00 | 70.00 | 64.00 | 60.00 | 60.00 | 62.00 | 60.00 | 62.00 | 62.00 | 66.00 | 62.00 | 63.00 | 50.00 | 1.00 | 6.00 | 5.00 | 3.00 | 2.00 | 3.00 | 4.00 | 5.00 | 4.00 |
| 2.00 | 28.00 | 66.00 | 2.00 | 15.00 | 4.00 | 110.00 | 112.00 | 110.00 | 106.00 | 110.00 | 100.00 | 105.00 | 100.00 | 98.00 | 90.00 | 90.00 | 96.00 | 95.00 | 94.00 | 96.00 | 60.00 | 70.00 | 70.00 | 69.00 | 70.00 | 60.00 | 67.00 | 68.00 | 70.00 | 60.00 | 60.00 | 70.00 | 65.00 | 60.00 | 66.00 | 116.00 | 118.00 | 110.00 | 108.00 | 110.00 | 100.00 | 98.00 | 90.00 | 86.00 | 80.00 | 80.00 | 78.00 | 79.00 | 78.00 | 76.00 | 100.00 | 1.00 | 8.00 | 6.00 | 3.00 | 2.00 | 6.00 | 5.00 | 4.00 | 4.00 |
| 2.00 | 35.00 | 63.00 | 2.00 | 12.00 | 4.00 | 110.00 | 115.00 | 105.00 | 110.00 | 115.00 | 113.00 | 103.00 | 103.00 | 100.00 | 101.00 | 97.00 | 97.00 | 100.00 | 98.00 | 100.00 | 70.00 | 70.00 | 67.00 | 72.00 | 70.00 | 60.00 | 60.00 | 56.00 | 60.00 | 61.00 | 58.00 | 58.00 | 60.00 | 60.00 | 70.00 | 110.00 | 108.00 | 100.00 | 102.00 | 110.00 | 100.00 | 96.00 | 97.00 | 90.00 | 89.00 | 85.00 | 80.00 | 80.00 | 81.00 | 80.00 | 150.00 | 6.00 | 20.00 | 6.00 | 3.00 | 4.00 | 4.00 | 6.00 | 3.00 | 3.00 |
